# *Tsc* Gene Locus Disruption and Differences in Renal Epithelial Extracellular Vesicles

**DOI:** 10.3389/fphys.2021.630933

**Published:** 2021-06-28

**Authors:** Prashant Kumar, Fahad Zadjali, Ying Yao, Brian Siroky, Aristotelis Astrinidis, Kenneth W. Gross, John J. Bissler

**Affiliations:** ^1^Department of Pediatrics, University of Tennessee Health Science Center and Le Bonheur Children’s Hospital, Memphis, TN, United States; ^2^Department of Clinical Biochemistry, College of Medicine & Health Sciences, Sultan Qaboos University, Muscat, Oman; ^3^Department of Molecular and Cellular Biology, Roswell Park Comprehensive Cancer Center, Buffalo, NY, United States; ^4^Department of Pediatrics, St. Jude Children’s Research Hospital, Memphis, TN, United States

**Keywords:** tuberous sclerosis complex, extracellular vesicles, miRNA, autophagy, cell signaling

## Abstract

In tuberous sclerosis complex (TSC), *Tsc2* mutations are associated with more severe disease manifestations than *Tsc1* mutations and the role of extracellular vesicles (EVs) in this context is not yet studied. We report a comparative analysis of EVs derived from isogenic renal cells except for *Tsc1* or *Tsc2* gene status and hypothesized that in spite of having similar physical characteristics, EVs modulate signaling pathways differently, thus leading to TSC heterogenicity. We used mouse inner medullary collecting duct (mIMCD3) cells with the *Tsc1* (T1G cells) or *Tsc2* (T2J cells) gene disrupted by CRISPR/CAS9. EVs were isolated from the cell culture media by size-exclusion column chromatography followed by detailed physical and chemical characterization. Physical characterization of EVs was accessed by tunable resistive pulse sensing and dynamic light scattering, revealing similar average sizes and zeta potentials (at pH 7.4) for EVs from mIMCD3 (123.5 ± 5.7 nm and −16.3 ± 2.1 mV), T1G cells (131.5 ± 8.3 nm and −19.8 ± 2.7 mV), and T2J cells (127.3 ± 4.9 nm and −20.2 ± 2.1 mV). EVs derived from parental mIMCD3 cells and both mutated cell lines were heterogeneous (>90% of EVs < 150 nm) in nature. Immunoblotting detected cilial Hedgehog signaling protein Arl13b; intercellular proteins TSG101 and Alix; and transmembrane proteins CD63, CD9, and CD81. Compared to *Tsc2* deletion, *Tsc1* deletion cells had reduced EV production and release rates. EVs from *Tsc1* mutant cells altered mTORC1, autophagy, and β-catenin pathways differently than EVs from *Tsc2*-mutated cells. Quantitative PCR analysis revealed the down regulation of miR-212a-3p and miR-99a-5p in EVs from *Tsc2*-mutated cells compared to EVs from *Tsc1*-mutant cells. Thus, EV-derived miR-212-3p and mIR-99a-5p axes may represent therapeutic targets or biomarkers for TSC disease.

## Introduction

Tuberous sclerosis complex (TSC) affects over one million people worldwide, and the disease manifests from abnormalities in embryonic and postpartum cell growth control that can impact every organ system. The *TSC* genes encode proteins that heterodimerize to repress mTORC1 activity. The *TSC1* gene encodes hamartin, and the *TSC2* gene encodes tuberin. Loss of either *TSC1* or *TSC2* locus function results in upregulated mTORC1 activity associated with disease (Nathan et al., 2017). Though excellent advances have been made in understanding the genetics and involvement of the mTORC1 pathway, how the disease manifestations develop is unclear.

Given that the TSC gene products interact to regulate the mTORC1 pathway, the human disease phenotype is unexpectedly different for patients with *TSC1*-associated disease compared to those with *TSC2*-associated disease (Dabora et al., 2001; Sancak et al., 2005; Aronow et al., 2012; Kothare et al., 2014). This genotype–phenotype difference is also observed in *Tsc* mouse models of neurological disease. *Tsc1*-associated disease is less severe than *Tsc2*-associated disease (Zeng et al., 2011; Magri et al., 2013), and evidence indicates this is not due to changes in mTORC1 activity (Magri et al., 2013). Murine renal solid tumor disease also exhibits a differential effect based on which *Tsc* gene is affected (Kobayashi et al., 2001). Loss of either gene leads to increased mTORC1 activity, but the mechanism for the disease severity difference is unknown.

Although a somatic mutation, or “second hit” mechanism, is involved in disease development, the steps leading to disease manifestation appears to be nuanced in organ systems like the brain and kidney. Central nervous system pathologies in TSC include cortical tubers, subependymal giant cell astrocytomas, and focal cortical dysplasia (Niida et al., 2001; Ramesh, 2003). Inconsistent with the somatic mutation mechanism of TSC, these lesions may lack identifiable somatic mutations (Jozwiak and Jozwiak, 2007), raising the question of whether these tumors contain a mixture of wild-type and *Tsc*-null cells (Au et al., 1999; Napolioni and Curatolo, 2008). The genomic heterogeneity of TSC lesions is further supported by the varied clonality within samples from angiomyolipoma, hamartoma, or tuber or subependymal giant cell astrocytoma (Niida et al., 2001; Ramesh, 2003). Analyses performed on tubers and microdissected giant cells showed equal expression of both TSC alleles; however, giant cells exhibited increased phospho-S6, indicating mTORC1 activation (Ramesh, 2003; Baybis et al., 2004), and rare giant cells within tubers have also lost TSC function (Crino et al., 2010). A possible explanation for this genotype–phenotype difference may involve extracellular vesicles (EVs). Patel et al. (2015) examined the impact of the loss of *Tsc1* on the function on surrounding normal cells using the *in vivo* mouse embryo-neural tube model. Based on their cell phenotype results, they posited that cells without *Tsc1* might secrete EVs that alter surrounding cells with a preserved *Tsc1* locus such that mutant cells disseminate the TSC disease phenotype. However, they did not isolate EVs (Patel et al., 2015).

To better understand renal cystic disease in TSC, we recently used immunofluorescence, immunohistochemistry, and fluorescent lineage-tracing experiments and identified a cell non-autonomous mechanism between TSC-deficient cells and TSC-intact cells (Bissler et al., 2019). This mechanism relies on EVs as the inductive signal and is reminiscent of the morphogen transport model involving primary cilia and left–right asymmetry [for perspective, see Vogel et al. (2010)]. EVs are known to be involved in normal renal collecting duct physiological adaption (Street et al., 2011; Oosthuyzen et al., 2016). The loss of the *Tsc2* gene in renal epithelial cells significantly increases EV production and alters the EV proteome (Zadjali et al., 2020). Thus, the role of EVs in renal development and physiology is becoming clearer. EVs are also involved in renal tubular changes in a von Hippel–Lindau zebrafish model (van Rooijen et al., 2018). Such a cell non-autonomous mechanism may explain why murine models of *Tsc* renal cystic disease fail to show a convincing “second hit” mechanism (Onda et al., 1999; Wilson et al., 2006) and human TSC-associated cysts robustly express hamartin and tuberin (Bonsib et al., 2016).

We reasoned that if the TSC disease involved EV signaling, we might be able to identify a difference in the effects of EVs based on the genetic mutation in the *Tsc* axis. Here we report the EV differences between *Tsc1* and *Tsc2* mutant renal collecting duct cells.

## Materials and Methods

### Cell Lines and Development of *Tsc1* Knockout Cell Lines

We used murine kidney-derived principal cell lines, i.e., mIMCD3, purchased from ATCC (ATCC^®^ CRL-2123^TM^, Manassas, VA, United States). We previously developed derivative isogenic cell lines by knocking out the *Tsc1* or *Tsc2* gene by CRISPR/Cas9, and the derivative lines were designated T1G and T2J, respectively (Bissler et al., 2019). The *Tsc1* gene was knocked out by targeting exon 4 (Bissler et al., 2019). Cystic kidney-derived epithelial cells, i.e., M1 cells, were also purchased from ATCC^®^ (CRL-2038^TM,^ Manassas, VA, United States). mIMCD3, T1G, and T2J cells were maintained in DMEM/F12 with 10% FBS, whereas M1 cells were maintained with DMEM/F12 with 5% FBS and supplemented with 5 μM dexamethasone. Cultures were maintained at 37°C in a humidified 95% air and 5% CO_2_ atmosphere.

### Protein Isolation and Western Blot

Proteins were isolated from cultured cells using Mammalian Protein Extraction Reagent (M-PER, Thermo Fisher Scientific #78501) with PhosSTOP (Sigma-Aldrich, 4906845001) and Complete protease inhibitor cocktail (Sigma-Aldrich, 11697498001) as per the manufacturer’s protocol. Isolated proteins were quantified using Bradford assay. Western blot analysis was done to identify EV-related protein markers. For this experiment, proteins isolated from EVs were separated by SDS-PAGE, followed by electrophoretic transfer of proteins to a nitrocellulose membrane. The membrane was blocked with 5% non-fat dry milk or 5% bovine serum albumin in TBST (Tris-buffered saline, 0.5% Tween 20) for 1 h. Then, the membrane was probed with an appropriate primary antibody and incubated for 16 h at 4°C, followed by incubation with horseradish peroxidase-conjugated secondary antibodies for 1 h at room temperature. The primary and secondary antibodies were diluted in 5% milk or 5% BSA from 1:00 to 1:1,000 and 1:5,000, respectively. The specific antibodies used for each protein (manufacturer, code, or clone number) as well as the specific concentration used for each antibody and whether milk or BSA was used as blocking agent can be found in Table 1.

**TABLE 1:** List of antibodies used in the study.

Antibodies	Manufacture	Catalog #	Clone #	Dilution	Blocking agent
Alix	Proteintech	12422-1-AP	Polyclonal	100	5% BSA
ARL 13B	Neuroma	75287020	N295B/66	500	5% MILK
CD63	MBL International	D263-3	R5G2	300	5% BSA
CD81	Cell Signaling	10037S	D5O2Q	1000	5% BSA
CD9	Invitrogen	10626D	Ts9	500	5% MILK
GAPDH	Proteintech	10494-1-AP	Polyclonal	10,000	5% BSA
p-S6K	Cell Signaling	2211S	Ser235/236	5000	5% BSA
S6K	Cell Signaling	2317S	54D2	1000	5% MILK
TSG101	Millipore sigma	MABC786	4A10	1000	5% MILK

### Isolation of Extracellular Vesicles by Column Chromatography

Extracellular vesicles were isolated from the conditioned media derived from mIMCD3, T1G, and T2J cells per our published protocol with minor modifications (Zadjali et al., 2020). Briefly, cells were grown in complete growth media, with 10% FBS up to 75–80% confluency. Then, the cells were washed three times with sterile phosphate-buffered saline (PBS) to remove any traces of serum and replaced with conditioned DMEM/F12 media without serum for 24 h. The isolated conditioned media were centrifuged at 2,000 x *g* for 20 min at 4°C to remove cells and debris. The supernatant was further loaded on a Millipore Amicon^®^ Ultra 15-ml 10K centrifugal filter (Cat# UFC901008, Burlington, MA, United States) and concentrated to 0.5 ml. The sample was applied to a qEV column (Izon Sciences, New Zealand) and immediately eluted with 0.5 ml of 0.2 μM filtered PBS. Initial fractions (1st–6th) were not expected to contain EVs and were thus discarded. The latter three fractions, i.e. 7th, 8th, and 9th, were considered EV-rich fractions and were collected as per the manufacturer’s instructions. Finally, these fractions were pooled together and concentrated up to 0.5 ml using an Amicon^®^ Ultra 2-mL Concentration device (Millipore).

### Characterization of EVs

#### Transmission Electron Microscopy

Extracellular vesicles were negatively stained and observed for size and surface morphology using a TEM. Briefly, 8 μl of freshly isolated EV suspension was dried on the top of a carbon mesh 200 grid at room temperature. The sample was then negatively stained by 2% uranyl acetate followed by washing with water to remove excess stain. The grids were visualized by TEM JEOL 2000EXII (JEOL, Peabody, MA, United States; access provided by the Neuroscience Institute, The University of Tennessee Health Science Center).

#### Tunable Resistive Pulse Sensing (TRPS) Analysis of EVs

Extracellular vesicles were further characterized by TRPS analysis using qNano Gold (Izon Sciences) as described by the manufacturer’s instructions. During this process, EVs were driven through a polyurethane nanopore (NP150, Part No #A54186) under a 15-millibar pressure and a voltage of 0.30 mV. Polystyrene beads CPC100 (Batch #B8748S) with a stock concentration of 1.2 × 10^13^ and a working dilution of 1:500 were used as a standard. EV samples (35 μl) were applied on top of the nanopore under the abovementioned conditions, and a minimum of 500 particle blockade signals were recorded. The data were processed and analyzed by Izon control suite v3.0.

#### Dynamic Light Scattering

The particle size distribution of EVs was examined using DLS. For analysis, EV samples (50 μl) were mixed with 950 μl milliQ water. The samples were loaded into a disposable polystyrene cuvette, and five measurements were recorded; the mean value of the measurements was documented.

### pH Effect on EV Zeta Potential

This experiment was performed to elucidate the effect of cyst microenvironmental pH on the zeta potential of EVs derived from cell lines. Equal numbers of EVs, i.e., 5 × 10^7^, derived from mIMCD3, T1G, and T2G cells, were incubated with 500 μL PBS at pH 7.4 (physiological pH) or pH 6.0 (renal cystic pH) for 15 min followed by zeta potential measurement by qNano. Initially, the qNano was calibrated using the standard beads (CPC100) and zeta potential was measured at three different applied voltages, i.e., 0.20, 0.18, and 0.16 mV, in the presence of a constant stretch for all measurements. The samples were measured at the same setting at either of the voltages as per manufacturer protocol. For each reading, a minimum of 200 particles were measured and the raw data were analyzed using Izon control suite v3.0.

### Release and Uptake of EVs

Equal numbers of mIMCD3 and T1G cells were transfected with eGFP-tagged CD63 (pcDNA3-EGFP, Addgene) using Lipofectamine plus reagent (Thermo Fisher Scientific, Waltham, MA, United States). Transfection efficiency was measured by calculating the ratio of the GFP signal to the nuclear stain DAPI signal. The images were captured using a Leica DMi8 fluorescence microscope (Leica, Germany). To measure eGFP-EV release, debris-free cell media were collected 48 h after transfection. Fluorescence intensity was measured at 480 nm (excitation) and 510 nm (emission). Mean fluorescence intensities were normalized to total protein in cell media. EVs were isolated from eGFP-CD63-transfected mIMCD3, T1G, and T2J cells and quantified. Equal numbers of EVs were then added to recipient cells (M1 cells) for 24 h to measure EV uptake as the number of GFP foci normalized to the number of DAPI-stained nuclei.

### Effect of EVs on mTOR and Autophagy

Briefly, 5 × 10^6^ M1 cells per well were cultured in DMEM/F12 serum-starved media in a 10-cm dish and treated with 50 × 10^6^ EVs derived from mIMCD3, T1G, or T2J cells for 24 h. Next, cell lysates were obtained, and western blots were performed using mTOR markers pS6-K and S6K. Western blots were quantified using ImageJ (NIH, Bethesda, MD, United States) and normalized to GAPDH.

### Total RNA Isolation From EVs and Transfection

Equal amounts of EVs (∼20 × 10^6^ particles) from mIMCD3, T1G, or T2J cells were used to isolate total RNA using a total exosomal RNA isolation kit (Invitrogen, Lithuania). For miRNA transfection, a Lipofectamine RNAiMAX transfection kit was used (Thermo Fisher Scientific). For control scramble transfection, mirVana^TM^ miRNA Mimic Negative Control was used (Thermo Fisher Scientific).

### RT-PCR and miRNA Analysis

Total cellular RNA was isolated from cells (miRNeasy Mini, Qiagen, Germany), and DNA was removed using DnaseI digestion. cDNAs were generated by reverse transcription (High Capacity cDNA Reverse Transcription Kit, Applied Biosystems, Foster City, CA, United States). SYBR Green-based real-time PCR was performed (PowerUp SYBR Master Mix, Thermo Fisher Scientific) with the following primer sequences; m_Rps6kb: GGTAAAGGGGGCTATGGAAA (forward) and GGTCCACAATGAAAGGGTGT (reverse), m_ @ Actin: CCAGTTGGTAACAATGCCATGT (forward) and CCAGTTGGTAACAATG-CCATGT (reverse). Results are expressed as a relative unit from the relative standard curve.

To predict the miRNA targeting mouse p70-S6K (Rps6kb1) mRNA, we followed a strategy similar to that performed on the human p70-S6K gene (Razaviyan et al., 2018). Here we used multiple algorithms and mouse miRNA search databases. The databases included miRBase, TargetScan, miRanda, miRDB, mIRtarBASE, DIANA-microT, PicTar, EIMMo3, and TargetS. All miRNAs from TargetScan prediction were tabulated, and matches from other databases were scored. miRNAs identified from three or more databases were selected. Selected miRNAs were aligned to mouse Rps6kb1 mRNA 5’ and 3’ untranslated regions (Utr) and coding sequences of isoform 1 (NM_001114334) and isoform 2 (NM_028259). Western blot analysis of mIMCD3 cells shows isoform 2 of the gene (*S6K*) with a protein band around 35 kDa. Therefore, we selected miRNAs targeting this isoform.

For miRNA quantification, total RNA was extracted from EVs of the three cell lines (total of 1 × 10^8^ particles) and 30 ng RNA was then reverse transcribed using a locked nucleic acid approach (miRCURY LNA RT Kit, Qiagen). Selected miRNAs were quantified using SYBR Green-based real-time PCR (miRCURY LNA SYBR Green PCR Kit, Qiagen) using a pre-designed primer assay from Qiagen. Expression was normalized using the global mean of three snRNA loading controls: *U6*, *Rnu1a1*, and *Rnu5g*. The relative fold changes in miRNA expression levels were calculated with the 2-ΔΔCt method.

### Statistical Analysis

All studies were performed in triplicate for all groups, and results are presented as mean ± standard deviation. Statistical analyses were done using Student’s *t*-test. Multiple values between groups were analyzed using ANOVAs. The level of statistical significance (*p*) was set at *p* < 0.05.

## Results

### *Tsc1* and *Tsc2* Deleted Renal Collecting Duct Cells Produce EVs *in vitro* Differentially

**FIGURE 1:**
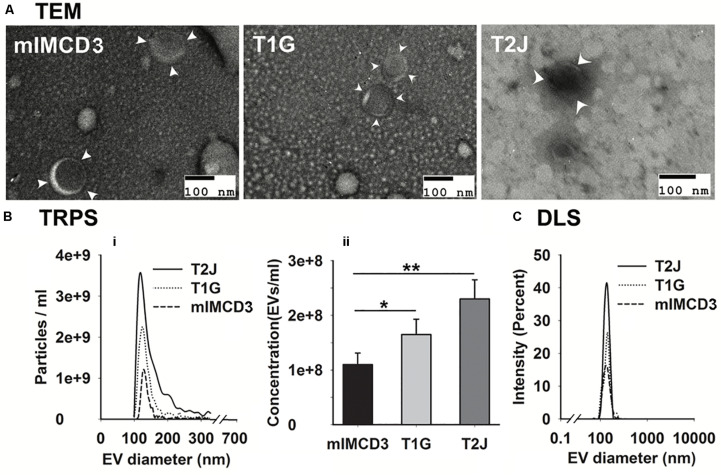
Characterization of mIMCD3, T1G, and T2J cells derived EVs. **(A)** Representation of transmission electron microscopy confirmed the rounded cup-shaped structure of EVs (indicated with white arrowhead) isolated from the cell culture media of all three cell lines (scale bar 100 nm). **(B-i,ii,C)** Tunable resistive pulse sensing (TRPS) and dynamic light scattering (DLS) analysis of purified EVs. Data (*n* = *3*) were represented as mean ± SD. *Significant difference analyzed by *t*-test (*p < 0.05, **p < 0.01).

Extracellular vesicles were isolated from the conditioned media of murine renal collecting duct cells (mIMCD3) and *Tsc1*-deleted mIMCD3 designated as T1G, as per our previous paper (Bissler et al., 2019). EVs were biophysically characterized using transmission electron microscopy (TEM; Figure 1A), tunable resistive pulse sensing (TRPS; Figure 1B-i), and dynamic light scattering (DLS; Figure 1C). The isolated EVs possessed a unique cup-shaped characteristic (Figure 1A) and heterogeneous size distribution which were similar to EVs derived from the *Tsc2*-deleted mIMCD3 cell line (Zadjali et al., 2020). TRPS showed a size of 123.5 ± 5.7 nm and 131.5 ± 8.3 nm for mIMCD3- and T1G-derived EVs, respectively. These EVs were in a similar size range as the EVs derived from the *Tsc2*-deleted mIMCD3 cell line at 127.3 ± 4.9 nm (T2J) (Zadjali et al., 2020).

**FIGURE 2:**
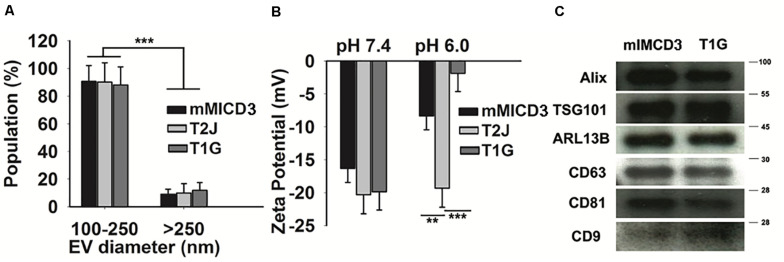
Particle population characteristics, zeta potential, and western blot analysis of cell-derived EVs. **(A)** Categorization of EV populations by size distribution by TRPS. The majority of EVs belong to a size range of 100–250 nm. *t-test* was performed between their counterparts. **(B)** pH affects the zeta potential of EVs: Compared to physiological pH (7.4), cyst microenvironmental pH (6.0) showed a drastic change in the zeta potential (measured by TRPS) of EVs derived from T1G cells. **(C)** Western blot analysis of EVs. Classical EV markers such as Alix, TSG 101, ARL13b, CD63, CD81, and CD9 were present in EVs derived from mIMCD3 and T1G cells. Data (*n* = *3*) were represented as mean ± SD. Significant difference analyzed by *t*-test (*p < 0.05, **p < 0.01, ***p < 0.001).

Our data suggest a significant increase in the mean EV production value derived from T1G (1.65 × 10^8^ ± 2.8 × 10^7^) and T2J (2.3 × 10^8^ ± 3.5 × 10^7^) as compared to that in the parental mIMCD3 (1.10 × 10^8^ ± 2.1 × 10^7^) cells (Figure 1B-ii). At physiological pH 7.4, the zeta potentials of mIMCD3-, T1G-, and T2J-derived EVs were recorded in a range of −15 to −20 mV (Figure 2B). At the renal cystic pH 6.0, the zeta potential of T1G-derived EVs and mIMCD3-derived EVs increased to −1.8 and −8.3 mV, respectively. There were no significant changes observed in the zeta potential for T2J-derived EVs. The majority of EVs from all three cell lines exhibited a similar small size (Figure 2A). To further verify that the isolated structures were EVs, we used western blot analysis to identify EV markers. Western blots of proteins isolated from the EVs identified the cellular proteins Alix and TSG101; the transmembrane proteins CD63, CD81, and CD9; and the cilia protein ARL13b (Figure 2C). We previously identified these EV markers in EVs from the *Tsc2*-deleted mIMCD3 cell line (Zadjali et al., 2020).

### Differences in EV Flux

**FIGURE 3:**
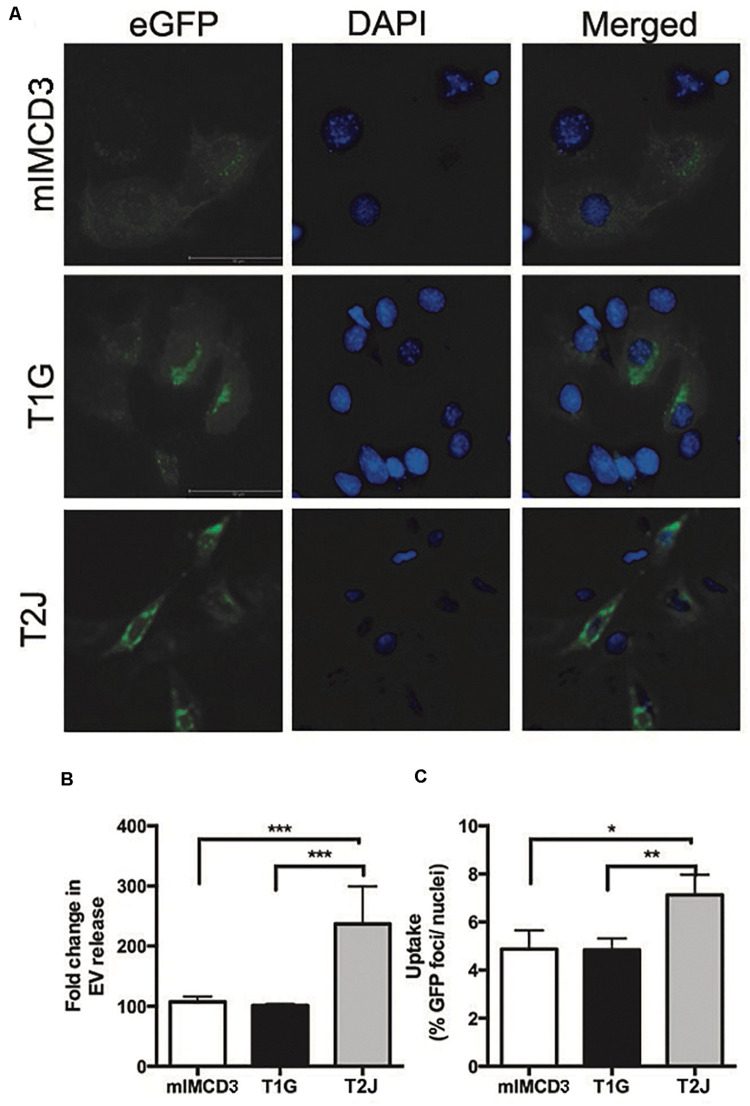
Release and uptake of extracellular vesicles. mIMCD3, T1G, and T2J cells were transfected with green fluorescent protein (eGFP)-tagged CD63, a marker of extracellular vesicles (EVs). **(A)** Immunofluorescence images of eGFP-transfected mIMCD3, T1G, and T2J cells with blue nuclear DAPI stain. **(B)** Fold difference in release in reference to mIMCD3 EV-release (*n* = 4) and **(C)** uptake of EVs from mIMCD3 (reference), T1G, and T2J cells. Cells were transfected with eGFP-CD63 plasmid. After 48 h, transfection media were collected, and cells were lysed. Mean fluorescence intensities (MFIs) normalized to protein content were quantified in cell media (to measure EV release). For uptake of GFP-positive EVs by intercalated M1 cell - EVs isolated from the culture media of mIMCD3, T1G, and T2J cells were used to treat M1 cells at a dose of 50 million EVs/ml in a microscopic slide chamber for 24 h. Slides were imaged, and the number of GFP positive foci was counted and normalized to the number of DAPI-positive nuclei. Mean ± SD data of total of 15–20 images were analyzed from two experiments. Student’s *t*-test was performed (*p < 0.05, **p < 0.01, ***p < 0.001).

To better understand how the *Tsc* gene status impacted the turnover of EVs, we examined EV release and uptake rates. We transfected mIMCD3, T1G, and T2J cells with a CD63-green fluorescent protein (GFP) construct and collected cell media to measure release post-transfection. This approach allowed us to assess EV production in the presence of fetal bovine serum (FBS). Experiments in Figure 1 were performed in the absence of FBS to avoid contamination with EVs from FBS. The transfection efficiency for the cell lines was equivalent (Figure 3A). The EV release rate (Figure 3B) was increased in the *Tsc2*-disrupted T2J cell line compared to that in the parental mIMCD3 cell line. T1G EVs had a release rate similar to that of mIMCD3 EVs. This is somewhat different from that seen in Figure 1, likely because in the absence of FBS, the cells were stressed and produced more EVs (Eguchi et al., 2020). We used EVs from these cells to treat intercalated M1 cells and measure the uptake rate. A higher uptake rate was observed with EVs from T2J cells, while T1G EV uptake was similar to mIMCD3 EV uptake (Figure 3C).

### *Tsc* Locus Modulates Different EV Effects on Target Renal Epithelial Cells

**FIGURE 4:**
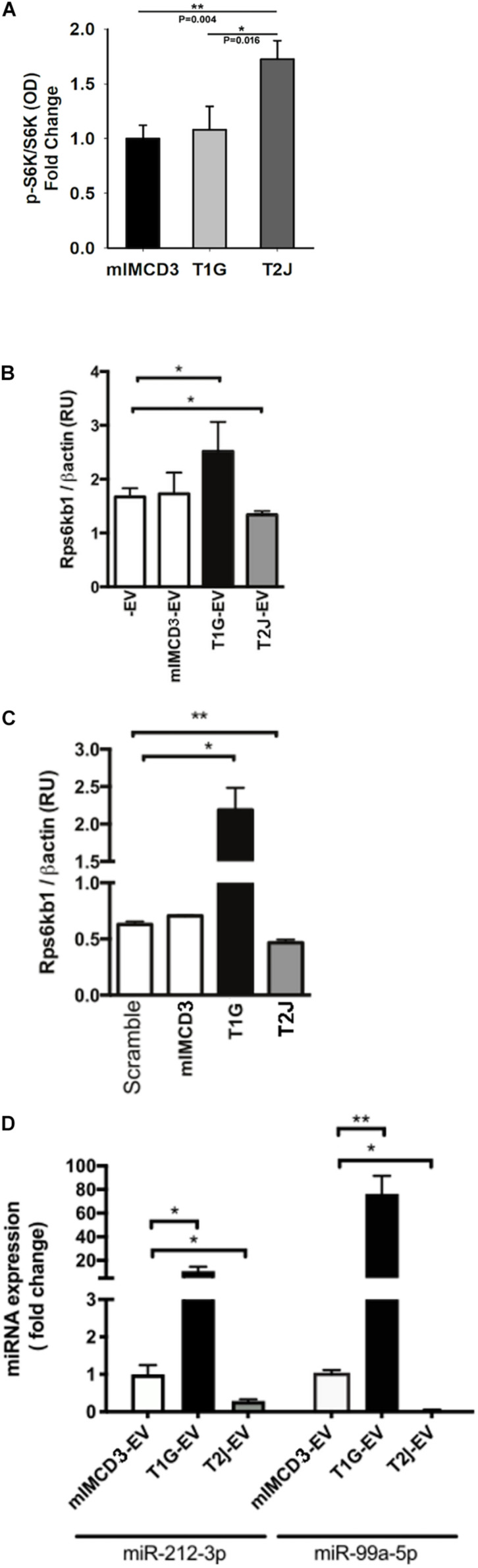
Cellular effects of EVs from *Tsc1*- or *Tsc2*-deleted cells on recipient cells. **(A)** Effect of EV treatment on mTORC1 signaling on the pS6K/S6K ratio. All values normalized to mIMCD3 and reported as fold change. **(B)** Effect of EV treatment on S6K mRNA (*Rps6kb1*) expression normalized to the expression of β-Actin. -EV means no EVs, and the cell type that produced the EVs is indicated. **(C)** Effect of total RNA (isolated from EVs) transfection on S6K mRNA expression. RNA extracted from mIMCD3, T1G, and T2J-EVs was transfected into serum-starved M1 cells. For the control, scrambled RNA was used (*n* = 3). S6K (*Rps6kb1*) gene expression was normalized to β-actin expression (RU: relative expression unit). **(D)** miRNA expression in mIMCD3, T1G, and T2J cells. Gene expression was normalized to the global mean of three loading controls. All experiments were performed in triplicate (*n* = 3), and statistical analysis was performed using one-way ANOVA followed by Tukey’s test (*p < 0.05, **p < 0.01).

Because the release and uptake rates are different for EVs derived from isogenic cell lines except for their *Tsc* gene status, we wished to understand if the different EV populations also had different effects on target collecting duct cells. We previously reported that EVs from *Tsc*-deleted cells induced more mTORC1 activity (S6K phosphorylation) (Bissler et al., 2019). Because EVs can transduce signals that affect transcription and translation, we wished to understand if this S6K effect was mediated by a change in gene expression. To assess this effect, we assayed M1 target cell RNA using RT-PCR normalized using *β-actin* after exposure to EVs. *RPS6KB1 (p70S6K)* mRNA expression was decreased in M1 cells treated with EVs from T2J cells but not from mIMCD3 cells (*p* < 0.05, Figure 4A). Paradoxically, EVs isolated from T1G cells induced an increased *RPS6KB1* mRNA expression, providing a further difference between the cell lines and their EVs at the RNA level, (p < 0.05, Figure 4A). These data suggest that EVs from T1G and T2J exert different effects and these differences may influence the phenotype severity between the genotypes.

### EV RNA Cargo Alter mTORC1 Activity in Recipient Cells

We have previously identified that when cells are exposed to EVs from the T2J cell line, the total S6K protein is decreased, while pS6K is increased, making the standard pS6k/S6K ratio change (Bissler et al., 2019). To investigate this further, we again exposed the M1 tubule cell line to EVs from the mIMCD3, T1G, and T2J cell lines and again observed the expected increase in the pS6K/S6K ratio for the T2J cell line and noted that the T1G and mIMCD3 lines did not exhibit this increase in pS6K and decrease in S6K that drives the ratio higher (Figure 4A). We wanted to see if the target M1 cell transcription had changed to help explain the change in the ratio. We used rtPCR to look at the transcriptional level for S6K protein and found that, in fact, the T2J cell line exhibited a reduced S6K gene expression, which was not observed in cells treated with EVs from the mIMCD3 (Figure 4B, *p* < 0.01). We also identified that the T1G cell line exhibited a significant increase in the S6K gene expression (Figure 4B), though protein levels are not different from the mIMCD3 cell line and the pS6K/S6K ratio is the same as mIMCD3 (Figure 4A).

Because EVs can transduce signals through proteins, mRNA, miRNA, lncRNA, and lipids, we wished to focus on the role of the EV RNA component. To investigate the role of RNA in these effects, we isolated EV RNA from ∼5 × 10^8^ EVs derived from mIMCD3, T1G, and T2J cell lines and used these RNA samples to transfect M1 cells. We used a commercially available scrambled RNA as a control and found that the RNA from the EVs seemed to drive the change in transcription for the *Tsc* mutant cell line. The T1G cell line exhibited an increase while the T2J cell line exhibited a decrease (Figure 4C).

### Extracellular Vesicle miRNA Targeting S6K Expression

The alterations in *RPS6KB1* gene expression upon transfection of EV isolated RNA derived from *Tsc* mutant T2J cell line EVs implicated an miRNA effect. Based on the changes we identified, we used the extracted total RNA from the EVs for quantitative PCR of the top 3 predicted miRNAs that target *RPS6KB1* mRNA. We identified three candidate miRNAs: miR-212-3p, miR-216b-3p, and miR-7116-5p. These miRNAs were most frequently identified by greater than three prediction tools. We also included a previously validated miRNA, miR-99a-5p, that reduces *RPS6KB1* gene expression (Tsai et al., 2018). miR-99a-5p and miR-212-3p expression levels were lower (*p* < 0.01) in EVs shed by T2J cells, but T1G-EVs induced a significantly higher expression of both miRNAs (Figure 4C). We failed to detect the expression of miR-216b-3p and miR-7116-5p in EVs.

## Discussion

Patients with *TSC2* mutations often have more severe disease manifestations than patients with *TSC1* mutations. Compared with *TSC1* deletion, *TSC2* deletion is linked to not only greater disease severity but also earlier symptom development (Kothare et al., 2014). Despite the significant progress in the understanding of TSC, a substantial gap in knowledge remains regarding why the disease severity is different depending on the locus involved. We previously reported that loss of the *Tsc2* gene resulted in renal cystic disease via a novel EV-mediated pathway (Bissler et al., 2019), and the mutant cells produced more EVs with an altered proteome (Zadjali et al., 2020). In this study, we hypothesize that the differences in EV characteristics, derived from *TSC1* and *TSC2* knocked out cells, may be dependent on the gene locus and thus affect the disease phenotype.

We isolated EVs from mIMCD3, T1G, and T2J conditioned (without serum, as serum contains an abundance of EVs) media by size-exclusion chromatography. Size-exclusion chromatography (SEC) is an easy-to-use and proven method to isolate EVs from a variety of clinical samples (Lobb et al., 2015). We preferred to use serum-free culture media instead of exosome-depleted media for EV isolation. Our cells (mIMCD3, T1G, and T2J) can tolerate and remain viable up to 48–56 h without serum, as assessed by cell viability MTT assay (data not included). Further, the column chromatography is a well-established and highly reproducible EV isolation method which elutes EVs with maximum purity and high yield (Lobb et al., 2015; Welton et al., 2015). To meet the criteria of the International Society for Extracellular Vesicles (ISEV), we characterized the EVs according to the standard guidelines (Théry et al., 2018). We further characterized EVs for their concentration and size distribution using TRPS and DLS. Although TRPS and DLS work on significantly different principles, both provided a similar size distribution, i.e., 123.5 ± 5.7 nm, 131.5 ± 8.3 nm, and 127.3 ± 4.9 nm, for mIMCD3-EVs, T1G-EVs, and T2J-EVs, respectively (Figures 1B,C).

The zeta potential of mIMCD3- and T1G-derived EVs at physiological pH (pH 7.4) was −16.3 ± 2.1 mV and −19.8 ± 2.7 mV, respectively. We observed a drastic drop in the zeta potential of T1G-EVs at cystic pH (pH 6.0) and interestingly no change in the zeta potential of T2J-EVs. Microenvironmental pH plays a very important role in EV trafficking. Generally, low pH facilitates EV release and uptake as described in melanoma cells (Parolini et al., 2009). The drop in zeta potential and pH could lead to a physical interaction between the vesicles: exosome–exosome interaction (“mitosis like” phenotype) (Beit-Yannai et al., 2018). These types of interactions are selective and might have an inhibitory effect on incoming signals, which could influence the EV-mediated signal modulation in various biological processes such as homeostasis and cancer metastasis (Beit-Yannai et al., 2018). Apart from physical characterization of EVs by TRPS, DLS, and TEM, western blot was performed to confirm the expression of EV-associated protein markers. As per the ISEV guidelines, EVs must be characterized for the presence of at least one transmembrane protein (CD63, CD81, or CD9) and one cytosolic protein (Alix or TSG101).

EVs can influence disease manifestation by regulating multiple signaling pathways. We found that EVs regulated mTORC1, and this pathway is known to regulate downstream Wnt/β-catenin and autophagy signaling pathways. We recently reported a cell non-autonomous involvement of mTORC1 in *Tsc* renal manifestation (Bissler et al., 2019; Barone et al., 2021). mTORC1 promotes the phosphorylation of an array of downstream effectors such as S6K, ULK1, TFEB, and 4EBP1, which further regulate various cellular processes such as cell proliferation, nucleotide synthesis, autophagy, and other growth-related signaling pathways (Zadjali et al., 2020). Furthermore, mTORC1 signaling cell autonomously suppresses Wnt/β-catenin signaling by promoting Dishevelled–clathrin AP-2 adaptor interaction and enhancing Dishevelled-mediated frizzled internalization (Zeng et al., 2018). In fact, the TSC1/TSC2 (hamartin/tuberin) complex can be associated with GSK3β and Axin and can therefore regulate the β-catenin degradation complex (Mak, 2003). In addition, autophagy plays a very crucial role in the development and homeostasis of normal tubular epithelial cells. Autophagy dysregulation is associated with renal manifestations including diabetic nephropathy, polycystic kidney diseases, acute kidney injury, and obstructive nephropathy (Huber et al., 2012; Kume et al., 2012). We posit that *Tsc*-mutant renal epithelial cells suppress autophagy and this feature may be involved in proliferation into cysts.

miRNA plays an important role in the development and progression of different types of cancer as well as in TSC (Volinia et al., 2006; Bagla et al., 2018). Identifying the responsible miRNA in TSC could lead to the development of novel biomarkers and therapeutic targets. In our current study, the qRT-PCR data showed the EV-mediated differential expression of miRNA-212-3p and miRNA-99a-5p. Deletion of the *Tsc1* gene leads to dramatically higher levels of miR-212-3p (approximately 20-fold change) compared to the control parental cell line (Figure 4D). By contrast, *Tsc2* gene deletion triggered the down regulation of miR-212-3p. Increased levels of miR-212-3p negatively regulate hepatocellular carcinoma by suppressing the connective tissue growth factor (Chen et al., 2019). miRNA-212 suppresses the X-linked inhibitor of apoptosis protein that constrains renal cancer invasion and proliferation, raising questions about a possible role in TSC-associated lymphangioleiomyomatosis. Furthermore, a low level of miR-212 is highly linked to T-stage and TNM-stage of renal cell carcinoma (Gu et al., 2017), which is related to cellular stress (Potter et al., 2017). In this study, we also identified changes in miRNA-99a-5p, which followed the same trend of expression as miRNA-212-3p. Compared to EVs from control mIMCD3 cells, EVs from *Tsc1* knockout cells facilitated a >80-fold increase in the expression of miRNA-99a-5p, whereas EVs from *Tsc2* knockout cells completely silenced the expression of miRNA-99a-5p. miRNA-99a-5p plays an important role in the pathogenesis of various types of cancer such as ovarian cancer (Yoshimura et al., 2018), oral cancer (Shi et al., 2017), and bladder cancer (Tsai et al., 2018). Downregulation of miRNA-99a-5p may contribute to the more severe disease phenotype in *TSC2-*associated disease than in *TSC1-*associated disease. For example, in bladder cancer tissue, overexpression of miR-99a-5p triggered the dual inhibition of the mTORC1 and mTORC2 signaling axis by the PI3K/AKT pathway (Tsai et al., 2018). Perhaps this activity may be leveraged to develop a combination therapy by combining miRNA-99a-5p and the mTOR inhibitor TSC in other cancers (Trelinska et al., 2016).

In conclusion, we demonstrate that EVs released from T1G and T2J cells possess different physical characteristics as well as different release and uptake rates. These differences in EV characteristics are due to the deletion of the *Tsc1* or *Tsc2* gene, and these differences may contribute to the differences in locus-associated disease severity. We demonstrate that the EVs from these isogenic cell lines except for their *Tsc* gene were responsible for the differential expression of the miR-212-3p/mTORC1 and mIR-99a-5p/mTORC1 axis and regulated S6K expression differently. These differences may impact TSC-associated cell proliferation. Our findings suggest that miRNA-212-3p and miRNA-99a-5p may be potential biomarkers and possible therapeutic targets for TSC disease.

## Data Availability Statement

The raw data supporting the conclusions of this article will be made available by the authors, without undue reservation.

## Author Contributions

FZ, JB, PK, and YY: conceptualization, methodology, investigation, and data curation. BS and JB: resources. AA, FZ, JB, KG, PK, and YY: writing—original draft preparation. AA, FZ, JB, KG, and PK: writing—review and editing. JB: supervision and funding acquisition. All authors contributed to the article and approved the submitted version.

## Conflict of Interest

The authors declare that the research was conducted in the absence of any commercial or financial relationships that could be construed as a potential conflict of interest.
